# Effects of Different Types of Stabilizers on the Properties of Foam Detergent Used for Radioactive Surface Contamination

**DOI:** 10.3390/molecules28166107

**Published:** 2023-08-17

**Authors:** Hao Zhang, Lili Liang, Hailing Xi, Xiaoyan Lin, Zhanguo Li, Yu Jiao

**Affiliations:** 1 School of Science, Xichang University, Xichang 615013, China; zhanghao1608@163.com (H.Z.); jiaoyu@xcc.edu.cn (Y.J.); 2 Engineering Research Center of Biomass Materials, Ministry of Education, School of Materials Science and Engineering, Southwest University of Science and Technology, Mianyang 621010, China; lily11311@163.com; 3 State Key Laboratory of NBC Protection for Civilian, Beijing 102205, China; lizhanguo@sklnbcpc.cn

**Keywords:** radioactive contamination, foam decontamination, different types of stabilizers, wettability, wall-hanging

## Abstract

Compared with high-pressure water and reagent washing decontamination, foam decontamination has a promising application due to its ability to significantly reduce the volume of radioactive waste liquids and effectively decontaminate the inner surface of the pipes, the interior of the large cavities, and the vertical walls. However, the foam is less stable, leading to a low decontamination rate. Currently, three main types of stabilizers with different stabilizing mechanisms, namely nanoparticles, polymers, and cosurfactants, are used to improve foam stability and thus increase the decontamination rate. Nanosilica (NS), xanthan gum (XG), and n-tetradecanol (TD) were used as typical representatives of nanoparticles, polymers, and cosurfactants, respectively, to improve the stability of the foam detergent with pH < 2 and chelating agents. The differences in the effects of these three types of stabilizers on foam properties were investigated. Although NS, XG, and TD all increase the half-life of the foam from 7.2 min to about 40 min, the concentration of TD is much lower than that of NS and XG in the foaming solution, and TD foaming solution has the highest foaming ratio. Moreover, TD can markedly lower the surface tension, resulting in a significant reduction of the wetting contact angle on the surfaces of glass, ceramic tile, stainless steel, and paint, while NS and XG cannot signally change the surface tension and have no obvious effect on the wetting contact angle. At low shear rates, TD can increase the apparent viscosity of foam by two orders of magnitude, and the wall-hanging time of the foam on the vertical wall is more than 30 min. In contrast, NS and XG cause a limited increase in the apparent viscosity of the foam, and the wall-hanging times are both less than 5 min. In addition, TD foaming solution has excellent storage stability, and the storage time has no obvious effect on the performance of the foam. And after only three days of storage, NS undergoes severe agglomeration and precipitation in the foaming solution, resulting in a complete loss of the stabilizing effect. After 90 days of storage, the half-life of XG foam decreases by 26%. For simulated radioactive uranium contamination on both horizontal and vertical surfaces, TD can significantly improve the decontamination rate, especially for vertical surfaces, where TD can increase the single decontamination rate by more than 50%.

## 1. Introduction

In order to achieve the “carbon peaking and carbon neutrality” goal, low-carbon, high-efficient, and clean nuclear power is being developed rapidly in China [1]. By 2021, China had the third largest nuclear power generation capacity in the world, behind only the United States and France [2]. It is expected that China’s nuclear power generation will rank first by 2030, and more than 100 nuclear reactors will be built [3]. However, nuclear power plants will inevitably produce radioactive contamination of surfaces during operation [4]. Moreover, the lifespan of nuclear power plants is usually 30 to 50 years, and the International Atomic Energy Agency estimates that about 200 nuclear reactors worldwide will be shut down between 2020 and 2040 [2], creating a large number of nuclear facilities contaminated by radionuclides. In addition, military nuclear facilities produce radioactively contaminated surfaces during operation and decommission. Therefore, it is necessary to decontaminate the polluted surface in time to prevent the radionuclides present on the surface from spreading into the air to form radioactive aerosols or migrating to water bodies [5].

There are decontamination methods such as mechanical [6,7], high-pressure water [6], laser [4,8], ultrasonic [9,10], reagent washing [11,12], electrochemistry [13], gel [5,14], and peelable film [15,16], each of which have advantages and drawbacks, as shown in Table S1. However, these decontamination technologies are difficult to achieve effective and low-cost decontamination when dealing with unconventional surfaces such as the inner surfaces of pipes and the interior of large cavities, vertical walls, and ceilings [5,17]. Compared with other decontamination technologies, foam decontamination has the following advantages. First, the volume of waste liquid is significantly reduced. The air in the foam occupies most of the volume fraction, and the amount of radioactive waste liquid is markedly lowered after defoaming, which signally decreases the cost of waste liquid treatment [5]. Secondly, the decontamination visualization is strong. The surfaces covered by foam are visibly white, which avoids repetition and omission and improves the accuracy of decontamination. In addition, the decontamination device is simple. A large amount of foam can be generated by using a home car-washing foamer. After the decontamination is completed, the foam can be recycled by using a high-powered vacuum cleaner [18]. Most importantly, the foam has excellent wall-hanging properties. After the single-phase foaming liquid is transformed into air–liquid two-phase foam, some physical and chemical properties will be abruptly changed, such as a significant decrease in density and a sharp increase in apparent viscosity, which can adhere to the inner surface of pipes, the interior of large cavities, vertical walls, and ceilings for a long time, prolonging the decontamination time of foam on these surfaces and thus improving the decontamination rate [5,17].

However, the foam is a thermodynamically and dynamically unstable system. Due to its high specific surface area and surface-free energy, the foam begins to rupture continuously after its formation to reduce the system energy, which eventually leads to the complete separation of the air and liquid that make up the foam [19], resulting in the complete loss of the unique physical and chemical properties of the foam. Therefore, improving the stability of the foam to maintain the characteristic physical and chemical properties is the key to enhancing the foam decontamination rate. Currently, nanoparticles [20], polymers [21], and cosurfactants [22] are mainly used to improve foam stability. The nanoparticles mainly depend on its adsorption at the air–liquid interface to increase the strength of liquid film and inhibit coalescence [20]. Polymers hinder drainage mainly by increasing the viscosity of the solution [21]. The cosurfactants mainly rely on increasing the adsorption density at the air–liquid interface to reduce the surface tension and inhibit coarsening and coalescence [22]. However, the differences have been rarely reported between three types of stabilizers with different stabilization mechanisms in regulating the decontamination foam performance. With pH < 2 and chelating agent, it is beneficial for radionuclides on the surface to be dissolved in the liquid carried by the foam [23,24]. In this study, 3-(N,N-dimethylmyristylammonio)propanesulfonate (NDMP), which has excellent foaming ability at low pH, is used as the foaming agent [17,24]. The chelating agent is phytic acid (PA), which also has a good chelating effect under strong acidic conditions [25]. NS is the most commonly used nanoparticle because of its excellent foam stabilization in strongly acidic environments [26]. The polymer is made of XG, which has a rigid main chain structure and can effectively improve foam stability in low pH environment [18]. The cosurfactant is TD, which has the same chain length as NDMP, and the head group structure is not affected by pH change [24]. In this paper, we mainly study the difference between the effect of NS, XG, and TD on the performance of decontamination foam. The chemical structures of NS, XG, TD, PA, and NDMP are shown in Figure S1.

## 2. Results and Discussion

### 2.1. Differences in Surface Tension and Solution Viscosity

**Figure 1:**
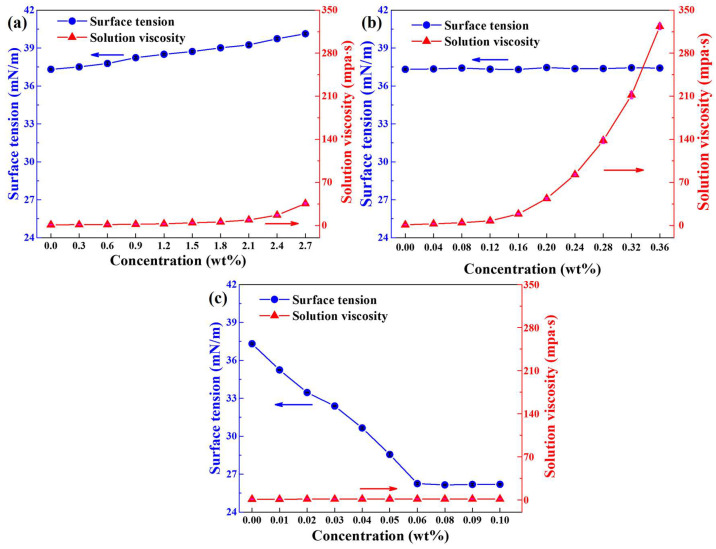
Surface tension and viscosity of the foaming solution as a function of (**a**) NS, (**b**) XG, and (**c**) TD concentration. The concentration of NDMP is 10.9 g/L, and the concentration of PA is 3.7 g/L.

The effects of NS, XG, and TD concentrations on the surface tension and solution viscosity were measured, respectively, as shown in Figure 1. The surface tension increases gradually with the increase of NS concentration. This is because the surfactant NDMP will be adsorbed on the NS surface, making the NS surface change from completely hydrophilic to moderately hydrophobic, so NS can also be adsorbed at the air–liquid interface, increasing the liquid film strength and improving foam stability [27,28]. However, NS and surfactant are competitively adsorbed at the air–liquid interface [29]. The increase of NS adsorption results in the decrease of surfactant adsorption at the air–liquid interface, leading to higher surface tension [30].

Solution viscosity rises slowly with the increase of NS concentration, and when NS concentration exceeds 2.1 wt%, the viscosity increases rapidly, as shown in Figure 1a. This may be similar to the polymer thickening mechanism. When the NS concentration is low, the viscosity increases mainly through the friction between nanoparticles and solvent molecules, and the viscosity gains very slowly. When the concentration exceeds a certain value, the NS are in contact with each other and the viscosity increases mainly through the mutual friction between nanoparticles, so the viscosity raises dramatically. The increase in solution viscosity helps to weaken the drainage and thus improve the foam stability [29].

The variation of XG concentration has little effect on the surface tension, as shown in Figure 1b. This is probably because XG does not interact with surfactants adsorbed at the air–liquid interface and thus does not affect the surface tension [21]. With the increase of XG concentration, the solution viscosity raises slowly at first and then rapidly. When the XG concentration is low, the critical overlap concentration is not reached [31], the XG molecules are not fully contacted, and the viscosity increase mainly depends on the mutual friction between XG molecules and solvent molecules, thus the viscosity does not gain markedly. When the XG concentration reaches the critical overlap concentration, the XG molecules are in contact with each other and the viscosity increases mainly by the mutual friction between XG molecules, so the solution viscosity increases significantly.

With the increase of TD concentration, the surface tension tends to decrease significantly and then remains unchanged, as shown in Figure 1c. This is because TD is a cosurfactant, which can adsorb at the liquid–air interface between foaming agent molecule, leading to increasing the adsorption density of the air–liquid interface and reducing the surface tension [32]. When the synergy adsorption at the air–liquid interface reaches saturation, the surface tension does not change. The solution viscosity hardly changes with the increase of TD concentration and is always low, closing to the viscosity of pure water, which helps to reduce the resistance during the foaming process and maintain high foaming properties. This will be further analyzed in Section 2.2.

### 2.2. Difference in Half-Life and Foaming Ratio

**Figure 2:**
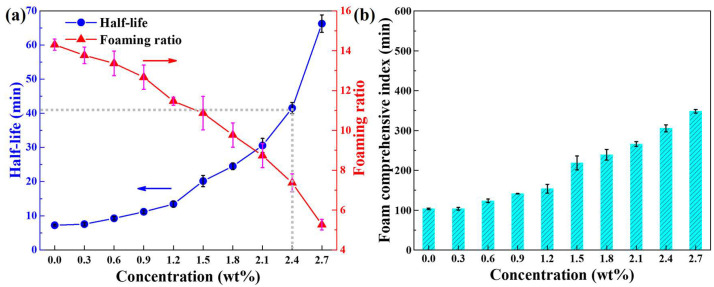
(**a**) Half-life, foaming ratio, and (**b**) foam comprehensive index as a function of NS concentration.

The influence of NS concentration on the foam stability and foamability is shown in Figure 2a. With the increase of NS concentration, the half-life representing the foam stability first increases slowly, and then grows rapidly when the concentration exceeded 1.2 wt%. This is mainly because when the NS concentration is low, there is not enough NS adsorbed at the air–liquid interface, and the foam film strength is low and easy to break [33], thus the foam stability is not high. When NS reaches a certain concentration, there are enough NS particles adsorbed at the air–liquid interface, the foam film strength is greatly improved, and the foam stability is significantly increased. The foaming ratio always decreases with the increase of NS concentration, because the surfactant will be adsorbed on the surface of NS, which reduces the free surfactant concentration in the solution, resulting in insufficient surfactant adsorbed at the newly formed air–liquid interface during the foaming process, leading to hindering the formation of foam [29].

It is worth noting that with the increase of NS concentration, the foam stability and foamability show an opposite trend, which means that the foam with high stability does not necessarily have good foaming properties. The foam comprehensive index [34] can be used to evaluate the overall performance impact of the stabilizer on the stability and foamability.

(1) $$C_{f}=T_{l}\cdot R_{f}$$

where *C_f_* represents the foam comprehensive index, min; *T_l_* is the half-life, min; *R_f_* is the foaming ratio. Figure 2b shows that the foam comprehensive index enhances with the increase of NS concentration.

**Figure 3:**
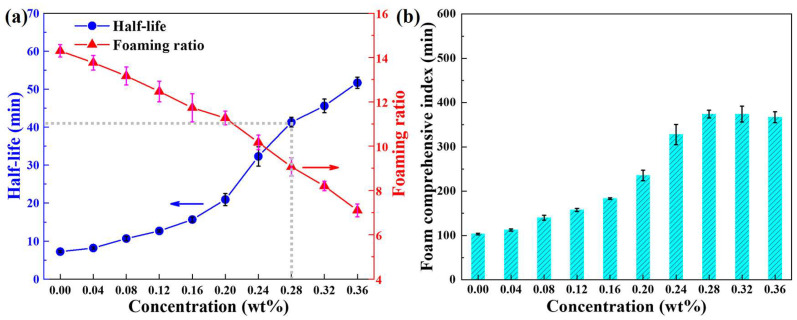
(**a**) Half-life, foaming ratio, and (**b**) foam comprehensive index as a function of XG concentration.

With the increase of XG concentration, the half-life tends to slowly increase at first and then rapidly increase, as shown in Figure 3a. XG mainly relies on increasing the viscosity of the solution to hinder the drainage, thereby improving the foam stability [18]. However, when the XG concentration is low, the critical overlap concentration is not reached, and the solution viscosity is low (Figure 1b), which does not have a significant hindering effect on the drainage, and thus the foam stability is poor. When the XG concentration reaches the critical overlap concentration, the XG molecules contact with each other, the solution viscosity increases significantly, the hindrance to the drainage enhances, and the stability of the foam also improves significantly. With the increase of XG concentration, the foaming ratio gradually decreased. This is mainly because the solution viscosity increases, which will magnify the resistance of the newly formed air–liquid interface in the foaming process, resulting in a reduction in the total area of the air–liquid interface with the same foaming energy, and thus the foam volume becomes smaller [35]. With the increase of XG concentration, the foam comprehensive index tends to firstly increase and then remains unchanged, as shown in Figure 3b.

**Figure 4:**
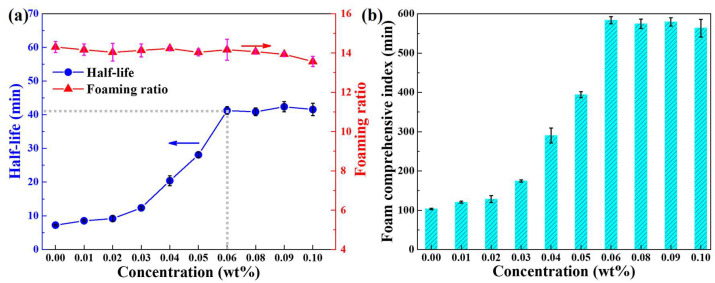
(**a**) Half-life, foaming ratio, and (**b**) foam comprehensive index as a function of TD concentration.

Unlike NS and XG, the foam half-life tends to increase at first and then remain unchanged with increasing TD concentration, while the foam ratio does not change much, as shown in Figure 4a. This is because TD will be adsorbed at the air–liquid interface together with surfactant, which increases the adsorption density and decreases the surface tension, thus slowing the coarsening and drainage, increasing the foam film strength and improving the foam stability [36,37]. When the adsorption at the air–liquid interface reaches saturation, the adsorption density no longer increases, the surface tension no longer decreases, and the hindering effect on drainage, coarsening, and coalescence reaches its maximum, so the foam stability no longer changes.

As the concentration of TD increases, the foaming ratio does not change markedly, because TD has little effect on the solution viscosity (the solution viscosity is close to that of water) and produces little resistance in the foaming process. Therefore, compared to XG foam, the foam volume does not decrease at the same foaming energy. With the increase of TD concentration, the foam comprehensive index tends to initially increase and then remain constant, as shown in Figure 4b.

Although NS, XG, and TD all improve the stability of the decontamination foam, their concentrations and foaming ratios are markedly different, as shown in Table 1. At a half-life of about 40 min, the concentration of TD is only 0.064 wt%, which is much lower than that of NS and XG, and the foaming ratio is also 1.91 and 1.55 times higher than that of NS and XG, respectively, and the comprehensive foam index is more than that of NS and XG.

For decontamination foam, the reduction of foaming ratio and the increase of solid content in a certain range are unfavorable, mainly reflected in the following two aspects: (1) The reduction of foaming ratio may result in lower wall-hanging property and shorter decontamination times on vertical and inclined surfaces, leading to lower decontamination rates. (2) The reduction of foaming ratio and the increase of solid content will increase the total amount of radioactive waste, thus increasing the cost of treatment [38].

Therefore, from the perspective of solid content and foamability, TD has a better application potential as a foam stabilizer in decontamination foam. In the following analysis, foams with a half-life of about 40 min were selected to study the effects of three different types of stabilizers (NS, XG and TD) on the wettability, wall-hanging, storage stability, and decontamination rate, and to further analyze the potential of different stabilizers for application in the field of surface radioactive decontamination.

### 2.3. Wettability Effect Analysis

**Figure 5:**
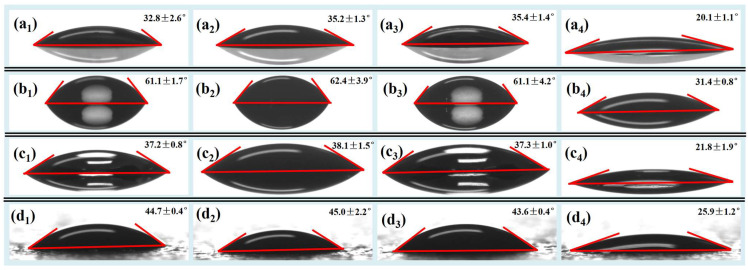
Wetting contact angles of (**a**) glass, (**b**) ceramic tile, (**c**) stainless steel, and (**d**) painted surfaces (The subscripts 1, 2, 3, and 4 represent ordinary, NS, XG, and TD foaming solutions, respectively). The concentrations of NS, XG, and TD are 2.4, 0.28, and 0.064 wt%, respectively.

The wettability determines whether the liquid carried by the foam can fully contact with the decontaminated surface, which has an important impact on the decontamination rate [18]. The wetting contact angles of ordinary, NS, XG, and TD foaming solutions were measured on glass, ceramic tiles, stainless steel, and painted surfaces, respectively, as shown in Figure 5.

On the same material surface, the wetting contact angle of TD foaming solution is markedly lower than that of other foaming solutions. The wetting contact angle of NS and XG foaming solution is not much different from that of ordinary foaming solution without a stabilizer, which can be explained by the Young equation [39].

(2) $$\gamma_{SA}-\gamma_{SL}=\gamma_{LA}\cdot \cos\theta$$

where *γ_SA_* represents the solid–air interfacial tension, mN/m; *γ_LA_* is the solid–liquid interfacial tension, mN/m; *γ_LA_* is the liquid–air surface tension, mN/m; *θ* represents the wetting contact angle, °. Equation (2) can be transformed into Equation (3).

(3) $$\cos\theta =\frac{\gamma_{SA}-\gamma_{SL}}{\gamma_{LA}}$$

On the same material surface, the solid–air interfacial tension *γ_SA_* is constant because the solid and air are not changed. The solid–liquid interfacial tension *γ_SL_* is difficult to be measured accurately, but its variation trend is often the same as that of the liquid–air surface tension *γ_LA_* [39]. It is found that *γ_LA_* is significantly reduced through the liquid–air surface tension test after the addition of TD, and thus *γ_SL_* is also markedly reduced. Compared with the ordinary foaming solution, it can be judged that cos *θ* increases significantly according to Equation (3), and thus the wetting contact angle *θ* decreases markedly. After adding NS and XG to the ordinary foaming solution, respectively, their *γ_LA_* did not change significantly (as shown in Figure 1a,b), so their *γ_SL_* did not change much either. Therefore, it can be judged that the cos *θ* of NS and XG foaming solution did not change much according to Equation (3), and thus their wetting contact angle *θ* did not change obviously.

Wettability measurement and analysis reveals that TD can cause a significant reduction in surface tension, leading to reducing the wettability contact angle on different material surfaces and improving the wettability of contaminated surfaces, which is conducive to the wetting and dissolution of surface contaminants and improving the foam decontamination rate.

### 2.4. Foam Rheology and Wall-Hanging Analysis

After the foaming solution is transformed into the foam, the rheological properties will change significantly, and the apparent viscosity will increase markedly and exhibit shear thinning behavior [30,40]. The foaming solution with high density and low viscosity is transformed into the foam with low density and high viscosity in the foam decontamination technology, which facilitates adhesion on inclined and vertical surfaces, extends the decontamination time on these surfaces, and improves the decontamination rate [17].

**Figure 6:**
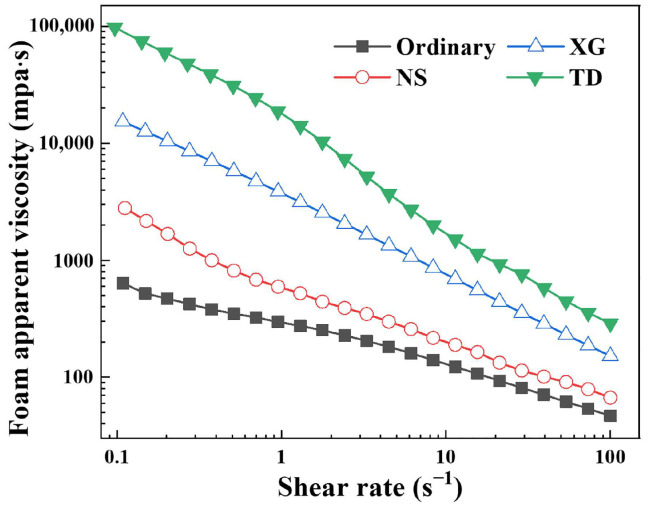
Foam apparent viscosity as a function of shear rate.

The apparent viscosity of different foams was measured by rheometer, as shown in Figure 6. The apparent viscosity of all foams decreases with increasing shear rate, showing a clear shear thinning characteristic. The apparent viscosity of different foams has distinct differences at the same shear rate, and the order of the foam apparent viscosity is always: TD foam > XG foam > NS foam > ordinary foam. However, the order of the foaming solution viscosity is: XG foaming solution (138.12 ± 4.85 mpa·s) > NS foaming solution (16.70 ± 1.12 mpa·s) > TD foaming solution (1.21 ± 0.03 mpa·s) ≈ ordinary foaming solution (1.04 ± 0.05 mpa·s). In other words, the viscosity of the foaming solution does not determine the apparent viscosity of the foam, and the foaming solution with low viscosity may form the foam with high apparent viscosity.

It can also be seen from Figure 6 that after adding stabilizers, the apparent viscosity of the foams increases, but the increase amplitude is not the same. The foam viscosity increase ratio is used to evaluate the viscosity enhancement effect of stabilizer on foam at the same shear rate. The calculation formula is shown as follows.

(4) $$F_{vi}=\frac{\eta_{i}-\eta_{O}}{\eta_{O}}=\frac{\eta_{i}}{\eta_{O}}-1$$

where *F_vi_* is the foam viscosity increase ratio, which characterizes the ability of the stabilizer to enhance the apparent foam viscosity. If *F_vi_* > 0, it indicates that the stabilizer can enhance the foam apparent viscosity. If *F_vi_* < 0, it indicates that the stabilizer can reduce the foam apparent viscosity. *η_i_* and *η_O_* are the apparent viscosity of the foam with and without stabilizer at the same shear rate, mpa·s, respectively. At the same shear rate, TD always has the largest foam increasing ratio, as shown in Table 2. As the shear rate increases, the foam viscosity increase ratio of all the stabilizers decreases. This shows that the stabilizer has a good thickening effect on the foam at low shear rate, especially TD, which can increase the apparent viscosity of the foam by more than two orders of magnitude at 0.1 s^−1^. At a high shear rate, the thickening effect of the stabilizer on foam decreases. The rheological properties of the stabilizers on foam are conducive to favorable engineering applications of foam, which are reflected in the following two aspects [24]: (1) In the process of converting foaming solution into foam by stirring method, there is often a high shear rate, and the foam stabilizer has no strong thickening effect on the foam, which is beneficial to the generation, transportation, and spraying of the foam. (2) After the foam is sprayed to the contaminated surface, the shear rate decreases and the thickening effect of the stabilizer raises, which enhances the resistance to the downward flow of the foam and facilitates the foam to adhere to the vertical and inclined surfaces, leading to extending the decontamination time and increasing the decontamination rate.

**Figure 7:**
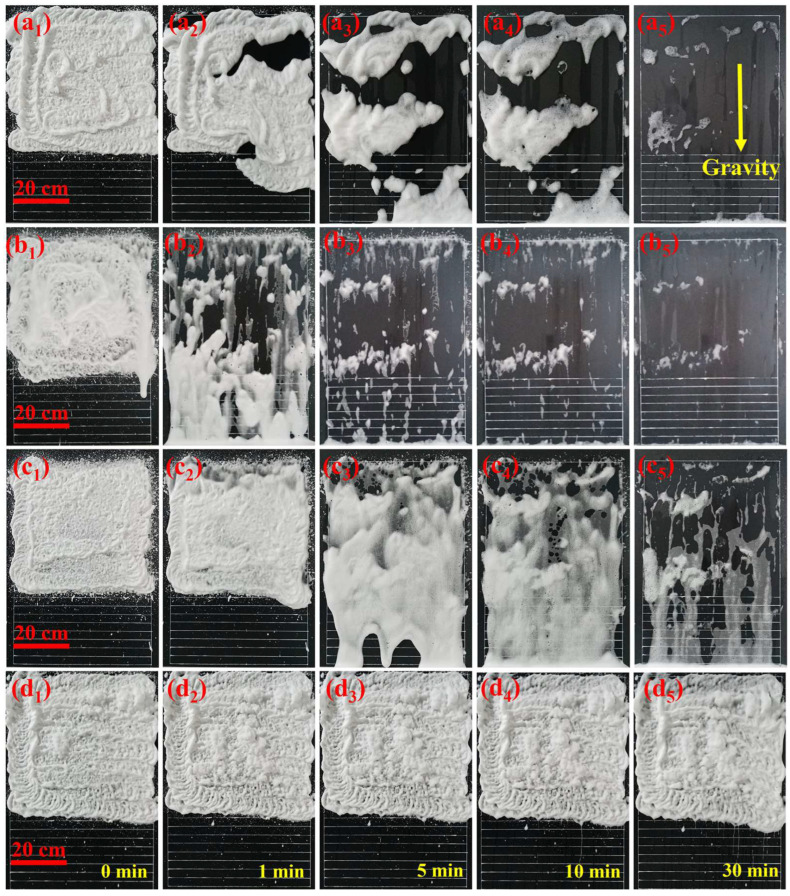
The flow of (**a**) ordinary foam, (**b**) NS foam, (**c**) XG foam, and (**d**) TD foam on the vertical wall (The subscripts 1, 2, 3, 4, and 5 represent 0 min, 1 min, 5 min, 10 min, and 30 min, respectively).

In order to determine the actual decontamination time of the foam on the vertical wall, the wall-hanging time of different foams were tested, as shown in Figure 7. The flow of foam on vertical wall was tested after spraying the foam to a 50 cm × 50 cm area by a foam gun. The spacing between adjacent test lines below the spraying area is 3 cm. The more foam flows through the test line in the same period, the less wall-hanging and the shorter the actual decontamination time at the vertical wall.

After the foam was sprayed to the vertical wall, NS foam flowed through all the test lines within 1 min, ordinary foam flowed through 4 test lines, XG foam flowed through only 1 test line, and TD foam did not flow. The order of foam wall-hanging properties is: TD foam > XG foam > ordinary foam > NS foam, which is different from the order of foam apparent viscosity (Figure 6). This is because the wall-hanging property of the foam is not only related to the foam’s apparent viscosity, but also to the foamability. If the apparent viscosity of the foam is the same, but the foamability decreases and the foam density increases, this is not conducive to the adhesion of the foam on the vertical surface. Although the apparent viscosity of NS foam is greater than that of ordinary foam, the foamability of NS foam is only 51.4% of that of ordinary foam, and the density of NS foam is nearly twice that of ordinary foam, resulting in the wall-hanging property of NS foam being less than that of ordinary foam.

Ordinary foam, NS foam, and XG foam all flowed through all test lines within 5 min, while TD foam did not flow. The TD foam hardly flows until 30 min, which indicates that the TD foam has excellent wall-hanging properties and can significantly extend the decontamination time on vertical walls, which is beneficial for improving the decontamination rate. This will be further analyzed in Section 2.7.

### 2.5. Mechanistic Analysis of the Effect of Stabilizers on Foam Properties

According to the variation rule of foam properties after NS, XG, and TD were added, the influence mechanism of the different stabilizers on foam properties was analyzed as follows.

**Figure 8:**
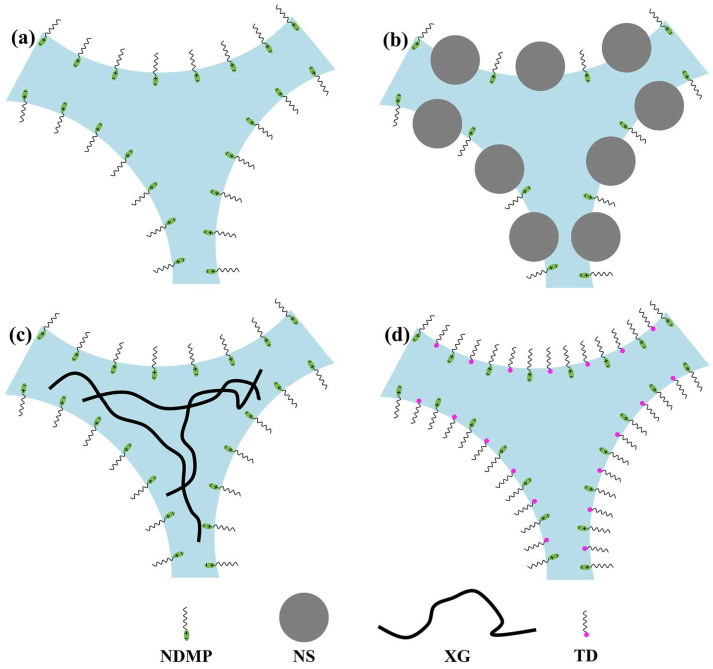
Schematic diagram including (**a**) only NDMP, and adding (**b**) NS, (**c**) XG, and (**d**) TD at the Plateau border.

In the ordinary foam without stabilizer, only NDMP is adsorbed at the gas–liquid interface, as shown in Figure 8a, foam film is easy to break, and the half-life is only 7.2 ± 0.2 min. Therefore, the stability of ordinary foam is very poor.

After NS is added, NS will be adsorbed at the gas–liquid interface, as shown in Figure 8b, thereby increasing the strength of foam film and preventing foam coalescence. Due to the large size of NS, the adsorbed NS increases the thickness of the gas–liquid interface significantly [20,41], and the impermeable NS also weakens the diffusion of gas, leading to retarding the coarsening. The half-life of the foam increased to 40.9 ± 1.6 min when the NS concentration was 2.4 wt%. However, the adsorption of NS and NDMP at the air–liquid interface is a competitive adsorption, and the increase of NS adsorption leads to the decrease of NDMP adsorption at the air–liquid interface. Therefore, the surface tension will increase slightly with the increase of NS concentration. In addition, a large amount of NDMP is adsorbed on the NS surface [28,42], which reduces the concentration of free surfactants in the solution, resulting in insufficient surfactant adsorbed at the newly formed gas–liquid interface in the foaming process [43], which hinders the formation of the foam. Therefore, as the concentration of NS increases, the NDMP adsorbed on the surface of NS raises, the concentration of free NDMP in the solution gradually decreases, and the foaming ratio gradually lowers.

After the addition of polymer XG, XG with longer molecular chains will form a mesh structure in the solution, as shown in Figure 8c, which increases the viscosity of the solution significantly and effectively inhibits the drainage of foam. When the XG concentration is 0.28 wt%, the half-life is 41.7 ± 1.3 min. However, the significant increase in solution viscosity enhances the resistance of the newly formed air–liquid interface in the foaming process, resulting in a decrease in the total surface area of the air–liquid interface at the same foaming energy. Therefore, with the increase of XG concentration, the viscosity of the foaming solution raises gradually, and the foaming ratio decreases gradually.

After adding the cosurfactant TD, TD will be adsorbed together with NDMP at the air–liquid interface to increase the adsorption density, as shown in Figure 8d, thereby improving the foam film strength [32] and preventing the coalescence between bubbles. The increase of adsorption density at the air–liquid interface will reduce the surface tension, which is conducive to reducing the capillary force and Laplace pressure difference, thus inhibiting the coarsening and coalescence [22,32,44]. When the TD concentration is 0.064 wt%, the adsorption at the air–liquid interface reaches saturation and the surface tension decreases from 37.32 ± 0.07 mN/m to 26.26 ± 0.09 mN/m, and the half-life increases to 41.2 min. With the increase of TD concentration, the viscosity of foaming solution basically does not change and is close to the viscosity of pure water, so it will not enhance the foaming resistance in the foaming process. Compared with NS and XG, the foaming ratio is less affected by the change of TD concentration.

### 2.6. Difference in Storage Stability

The foaming solution should be stored for a long time, as the preparation of foaming solution may miss the optimal disposal time during the nuclear emergency decontamination process. Therefore, the storage stability of the foaming solution is an important factor to determine whether it can be applied.

**Figure 9:**
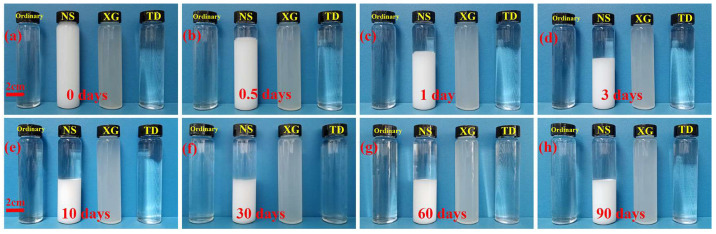
Foaming solutions at storage of (**a**) 0 days, (**b**) 0.5 days, (**c**) 1 day, (**d**) 3 days, (**e**) 10 days, (**f**) 30 days, (**g**) 60 days, and (**h**) 90 days.

With the increase of storage time, the ordinary, XG, and TD foaming solution were always free of precipitation and stratification, as shown in Figure 9. When NS foaming solution was stored for only 0.5 days, there was an obvious stratification phenomenon, NS gradually gathered at the bottom, and the upper part of the solution became clear. After 10 days of storage, the volume of NS aggregated at the bottom no longer changes. The aggregation and precipitation of NS in solution is mainly due to the small particle size of NS, which has a high specific surface area and is highly susceptible to agglomeration [45]. After multiple NS are aggregated together, the density of the agminated NS continually increases. Therefore, NS is gradually deposited to the bottom by gravity, and NS cannot be suspended in solution for a long time.

**Figure 10:**
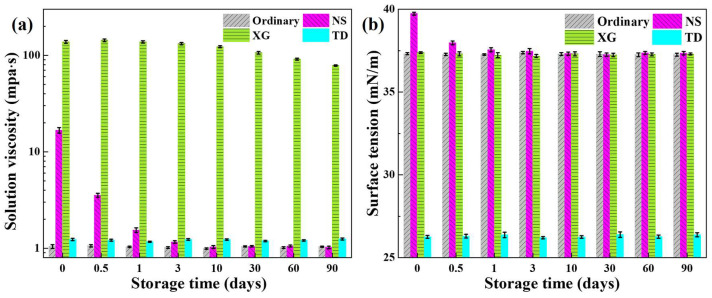
(**a**) Solution viscosity and (**b**) surface tension as a function of storage time.

The viscosity of the foaming solution was measured with storage time, as shown in Figure 10a. With the increase of storage time, the viscosity of the ordinary and TD foaming solution was almost unchanged and was slightly higher than that of pure water. The viscosity of NS foaming solution decreases rapidly, and the viscosity of NS foaming solution is similar to that of ordinary foaming solution after three days of storage, which is due to the agglomeration and precipitation of NS particles, leading to reducing the content of NS in the upper solution. The viscosity of XG foaming solution tends to decrease slowly with the increase of storage time, which may be that the microorganisms degrade XG in the foaming solution [46,47], leading to the reduction of XG molecular weight.

The storage time has a minimal effect on the surface tension of the ordinary, XG, and TD foaming solution, as shown in Figure 10b. The surface tension of NS foaming solution decreases rapidly within three days of storage, and then changes minimally. This is because the NS agglomeration and precipitation reduces the NS content in the foaming solution, resulting in insufficient NS adsorption at the gas–liquid interface, causing the adsorption sites to be “occupied” by the surfactant NDMP again, thus lowering the surface tension.

**Figure 11:**
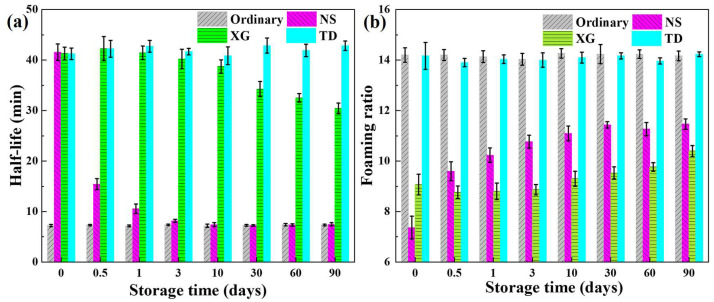
(**a**) Half-life and (**b**) foaming ratio as a function of storage time.

With the increase of storage time, the half-life of different foams shows various change rules, as shown in Figure 11a. The half-life of the ordinary foam changes little over 90 days, indicating that storage time has no effect on the foam stability of the surfactant.

The half-life of NS foam changes very markedly with increasing storage time. After 0.5 days of storage, the half-life of the NS foam decreases from 40.9 ± 1.6 min to 15.4 ± 1.1 min. After three days of storage, the half-life of NS foam is similar to that of ordinary foam, indicating that the stabilizing effect of NS on foam is almost completely lost. This is mainly due to the serious agglomeration and precipitation of NS in the foaming solution, resulting in insufficient NS adsorbed at the gas–liquid interface to stabilize the foam. The half-life of XG foam tends to decrease with increasing storage time. After 90 days of storage, the half-life of XG foam reduces from 41.7 ± 1.3 min to 30.4 ± 1.0 min. This is caused by the gradual decrease in the XG foaming solution viscosity, leading to an increase in the drainage.

The stability of TD foam is almost not affected by the storage time, and the half-life of TD foam is always around 40 min within 90 days. This is due to the fact that the adsorption density of TD at the gas–liquid interface does not change much, and the surface tension is always low and does not change significantly, which has a strong inhibitory effect on the drainage, coarsening and coalescence.

With the increase of storage time, the foaming ratio of different foams also showed various changes, as shown in Figure 11b. The foamability of ordinary and TD foam is not affected by storage time and has the highest foaming ratio. The foam ratio of NS foam increased first, and the foam ratio does not change much after the storage time reaches 10 days. The foaming ratio of NS foam increases with the storage time, which may be due to the release of some surfactant molecules adsorbed on the surface of NS after NS agglomeration. This increases the concentration of free surfactant molecules in the solution, enhances the number of surfactant molecules adsorbed on the newly formed air–liquid interface in the foaming process, and thus raises the foaming ratio. The half-life of XG foam tends to increase with the increase of storage time because the viscosity of XG foam decreases, resulting in reducing the resistance in the foaming process.

### 2.7. Difference in Decontamination Performance

**Figure 12:**
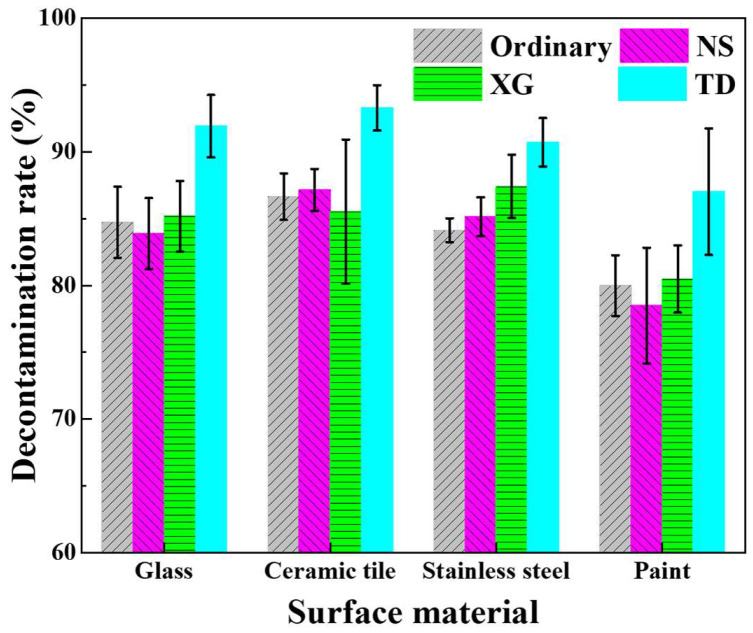
Decontamination rate of simulated radioactive uranium contamination on the horizontal surfaces.

The decontamination performance of the foam is not only affected by the stabilizer, but also by the surface material, the way the surface is placed, and the decontamination times [48]. The decontamination rates of ordinary, NS, XG, and TD foam were measured for simulated radioactive uranium contamination on horizontal surfaces of different materials within a decontamination time of 30 min, as shown in Figure 12.

On the same material surface, the decontamination rate of ordinary, NS, and XG does not differ much, while the decontamination rate of TD foam is obviously higher than other foams. This may be due to the fact that the wetting contact angle of TD foam is significantly smaller than that of other foams (Section 2.3), which enables the radioactive contaminants to be wetted sufficiently and dissolved better in the foam, thus improving the decontamination performance. For the same foam, the decontamination rate on the paint surface is slightly lower than that of other materials, probably due to the greater surface roughness of the paint (Table 3), leading to the radioactive contaminants potentially binding more firmly on the paint.

**Figure 13:**
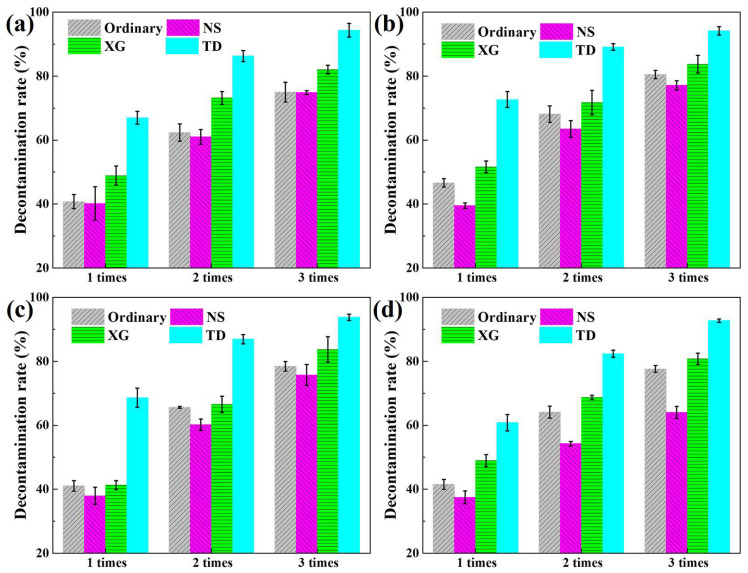
Decontamination rates of radioactive uranium contamination on vertical (**a**) glass, (**b**) ceramic tile, (**c**) stainless steel, and (**d**) painted surfaces.

The decontamination performance of the ordinary, NS, XG, and TD foam was tested on vertical surfaces within 30 min, as shown in Figure 13. The decontamination rate of TD foam is significantly higher than other foams for the same surface material and decontamination times. This is because the wall-hanging time of TD foam on vertical surfaces is more than 30 min, while the wall-hanging time of its ordinary, NS, and XG foam is less than 5 min. The actual decontamination time of TD foam is 30 min, so the decontamination rate of TD foam is higher than that of other foams. However, for the same foam, the single decontamination rate on the vertical surface is lower than that on the horizontal surface. This is due to the fact that for horizontal surface decontamination, the foam is always in contact with the radioactive contaminant, while for vertical surface decontamination, the contact time between the normal, NS, and XG foams and the vertical wall surface is signally reduced, thus decreasing the single decontamination rate. For TD foam, although it is always in contact with the vertical surface, TD foam will have the continuous drainage in the vertical direction, resulting in a decrease in the liquid content of the foam, leading to a reduction in the solubility of radioactive contaminants. Therefore, the decontamination rate of TD foam also declines on the vertical surface.

In order to improve the decontamination rate of the foam on the vertical surface, multiple decontamination can be carried out [48]. As decontamination times increase, the decontamination rate of all foams raises, as shown in Figure 13. However, after three times of decontaminations on vertical surfaces, only TD foam has a decontamination rate higher than 90% on glass, ceramic tile, stainless steel, and painted surfaces.

## 3. Materials and Methods

### 3.1. Materials

Foaming agent: 3-(N,N-dimethylmyristylammonio)propanesulfonate (NDMP, purity > 98%) was purchased from Aladdin Biochemical Technology Co., Ltd. (Shanghai, China). Stabilizers: nanosilica (NS, 15 ± 5 nm, purity > 99.5%), xanthan gum (XG, USP), and n-tetradecanol (TD, purity > 98%) were supplied by Aladdin Biochemical Technology Co., Ltd. (Shanghai, China) and Macklin Biochemical Technology Co., Ltd. (Shanghai, China), respectively. Chelating agent: phytic acid solution (PA, 50% in H_2_O) was purchased from Aladdin Biochemical Technology Co., Ltd. (Shanghai, China). Radioactive contaminants: Uranyl nitrate (UN, purity > 95%) was supplied by Chushengwei Chemical Co., Ltd. (Wuhan, China).

### 3.2. Methods

#### 3.2.1. Preparation of Different Foaming Solutions

NDMP was dissolved in deionized water to a concentration of 10.9 g/L (about 30 mM).

NS was added to the NDMP solution so that its concentration was 0~2.7 wt %, and then the NS was fully dispersed in the solution by ultrasound for 120 min. After stirring for 3 h at 20 °C with a magnetic stirrer, the pH of the solution was adjusted to 1.8 by adding PA. Following that, the solution was continuously stirred to prevent NS agglomeration and precipitation.

The NDMP solution was first stirred by the magnetic stirrer, and then XG was slowly added to prevent XG from aggregating to form large gel clusters, so that the XG concentration was 0 to 0.36 wt%. Following that, the temperature of the solution was adjusted to 50 °C, and the solution was stirred for 3 h. After XG was fully dissolved, the pH of the solution was adjusted to 1.8 by adding PA. Finally, the prepared XG foaming solution was stored in the incubator at 20 °C.

TD was added to the NDMP solution to the concentration of 0–0.0965 wt%, and the solution was stirred at 60 °C for 3 h by a magnetic stirrer. The solutions were stirred at 20 °C for 2 h, and the pH of the solution was adjusted to 1.8 by adding PA. Finally, the prepared TD foaming solution was stored in the incubator at 20 °C.

#### 3.2.2. Half-Life and Foaming Ratio Tests

50 mL of foaming solution was fully foamed by the agitator (EW-071, Zheguang Precision Co., Ltd., Dongguan, China) for 1 min at 12,000 r/min. The foam was then quickly poured into the measuring cylinder, and the volume of the foam was recorded as *V_f_*. If there was foam attached to the wall of the beaker, a medicine spoon was needed to scoop the foam from the wall of the beaker into the measuring cylinder. The time for foam to produce 25 mL liquid in the measuring cylinder was the half-life *T_l_*, which was used to evaluate the stability of the foam. In order to eliminate the influence of different volume, the foaming ratio was used to evaluate the foamability of the solution, as shown in the following equation.

(5) $$R_{f}=\frac{V_{f}}{V_{l}}$$

where *R_f_* represents the foaming ratio; *V_f_* is initial foam volume, ml; *V_l_* is the volume of the foaming solution, ml. The half-life and foaming ratio of the foaming solution were obtained by averaging the measured value of three independent measurements, and the test temperature is 20 ± 3 °C.

#### 3.2.3. Surface Tension Test

The surface tension of the foaming solution was measured by the multipurpose tensiometer (SIGMA 700, KVS Instruments Ltd., Helsinki, Finland) using the Du Noüy ring method. The surface tension was obtained by averaging the measured value of three independent measurements at 20 ± 1 °C.

#### 3.2.4. Foaming Solution Viscosity Test

The viscosity of the foaming solution was measured using the rotational viscometer (NDJ-5S, Pingxuan Scientific Instrument Co., Ltd., Shanghai, China) at 20 ± 1 °C. The speed of the viscometer was 60 r/min, and the suitable rotor type was selected so that the range percentage was between 10% and 90%. The viscosity of the foaming solution was obtained by averaging the measured value of three independent measurements.

#### 3.2.5. Wetting Contact Angle Test

The wetting contact angles of ordinary, NS, XG, and TD foaming solutions on glass, ceramic tile, stainless steel, and painted surfaces were measured at 20 ± 3 °C by the contact angle tester (K100, Krüss Scientific Instruments Ltd., Hamburg, Germany).

#### 3.2.6. Foam Rheology Test

The foaming solution was fully foamed for 1 min at 12,000 r/min by the agitator, and 2 mL of the foam was evenly applied to the measurement table. The apparent viscosity of the foam was measured from 0.1 s^−1^ to 100 s^−1^ at 20 °C by the rotary rheometer (HAAK MARSII, Thermo Fisher Scientific Ltd., Bremen, Germany) with the C60/2Ti rotor.

#### 3.2.7. Foam Wall-Hanging Performance Test

A 50 cm × 50 cm spraying area was drawn on the vertical surface, and a test line was drawn at 3 cm intervals directly below the spraying area. At the same time, the more test line foam flowed through, the worse the wall-hanging performance. The output pressure of 0.7 MPa was generated by the air compressor (V-0.17, Lida Machinery Co., Ltd., Quanzhou, China), and the decontamination foam was applied to the delimited 50 cm × 50 cm area by a foam gun (SG-GC024, Xinge Co., Ltd., Shanghai, China). The flow of the foam was recorded by a video camera for 30 min.

#### 3.2.8. Storage Stability Test

The ordinary, NS, XG, and TD foaming solution 1 L were prepared and sealed in a conical bottle at 20 °C. 50 mL of the upper solution was taken at 0, 0.5, 1, 3, 10, 30, 60, and 90 days, and the half-life, foaming ratio, surface tension, and solution viscosity were measured, respectively.

#### 3.2.9. Simulated Radioactive Uranium Decontamination Experiment

The plate surface with 10 cm × 10 cm was washed with deionized water and dried, and then the surface roughness of the plate was measured by a surface roughness meter (TR100, Beijing Jitai Keyi Testing Instrument Co., Ltd., China). A contamination area of 5 cm×5 cm was divided in the center of the plate, and the background count rate L_0_ of the test plate surface was measured by surface radioactive contamination measuring instrument (FJ-2207, Xi’an Nuclear Instrument Ltd., Xian, China). After 1 mL of uranium contamination solution was evenly smeared 5 cm × 5 cm and then dried by an electric heating plate at 35 °C, the count rate L_1_ of the simulated radioactive pollution was measured. The foam was sprayed onto the contaminated plates by the foam gun. After 30 min, the foam was sucked away by the foam recovery device. After drying on an electric heating plate at 35 °C, the count rate L_2_ was measured after decontamination. According to Equation (6), the foam decontamination rate was calculated.

(6) $$D_{R}=\frac{\left(L_{1}-L_{0}\right)-\left(L_{2}-L_{0}\right)}{L_{1}-L_{0}}\times 100\%=\frac{L_{1}-L_{2}}{L_{1}-L_{0}}\times 100\%$$

where *D_R_* is the decontamination rate, %; *L*_0_ is the background count rate before contamination; *L*_1_ is the count rate after contamination; *L*_2_ is the count rate after decontamination.

## 4. Conclusions

The effects of different types of stabilizers on the properties of decontamination foam were studied in this paper. Although the three types of stabilizers NS, XG, and TD can all improve the stability of the foam, the half-lives of the foams increased from 7.2 min to about 40 min, the concentration of NS, XG, and TD is 2.4 wt%, 0.28 wt%, and 0.064 wt%, and the foaming ratio of NS, XG, and TD is 7.4 ± 0.45, 9.1 ± 0.41, and 14.1 ± 0.53, respectively. TD as the stabilizer is beneficial to reduce the amount of waste liquid after defoaming and the solid content after volume reduction. NS and XG do not markedly change the surface tension and have no significant effect on the wetting contact angle. TD dramatically reduces surface tension, resulting in significantly lowering wetting contact angles on surfaces such as glass, ceramic tile, stainless steel, and paint. At a low shear rate, TD can increase the foam apparent viscosity by 152.1 times, while NS and XG can only increase by 23.6 times and 3.4 times, respectively. TD can signally increase the foam flow resistance. The wall-hanging time of TD foam is greater than 30 min on the vertical surface, while the wall-hanging time of both NS and XG foams was less than 5 min. In addition, TD foaming solution has good storage stability, and the foam properties do not change after 90 days of storage. However, due to the serious aggregation and precipitation of NS in the foaming solution, the foam stabilization effect of NS is lost after three days of storage. With the increase of storage time, the viscosity of XG foam decreases gradually, and the foam half-life decreases. Because TD foam can significantly improve the wettability and wall-hanging, the decontamination rate of simulated radioactive uranium contamination is improved on both horizontal and vertical surfaces, especially on vertical surfaces, where TD can increase the single decontamination rate by more than 50%.

## Figures and Tables

**Table 1:** The foam stabilizer concentration, foaming ratio and foam comprehensive index with a half-life of about 40 min. The concentration of NDMP is 10.9 g/L, and the concentration of PA is 3.7 g/L.

Foam Stabilizer	Concentration (wt%)	Foaming Ratio	Foam Comprehensive Index (min)	Surface Tension (mN/m)	Foaming Solution Viscosity (mpa·s)
NS	2.40	7.4 ± 0.45	305.4 ± 8.9	39.74 ± 0.09	16.70 ± 1.12
XG	0.28	9.1 ± 0.41	374.0 ± 8.6	37.38 ± 0.05	138.12 ± 4.85
TD	0.064	14.1 ± 0.53	583.8 ± 9.2	26.26 ± 0.09	1.21 ± 0.03

**Table 2:** Foam viscosity increase ratio at different shear ratio.

Foam Stabilizer	0.1 s^−1^	1 s^−1^	10 s^−1^	100 s^−1^
NS	3.39	0.99	0.54	0.43
XG	23.06	11.97	4.64	2.25
TD	152.07	62.18	11.28	5.17

**Table 3:** Surface roughness of different materials.

Surface Material	Glass	Ceramic Tile	Stainless Steel	Paint
Surface roughness (μm)	0.01 ± 0.01	0.10 ± 0.03	0.08 ± 0.02	0.61 ± 0.11

## Data Availability

Data is contained within the article.

## Supplementary Materials

The following supporting information can be downloaded at: https://www.mdpi.com/article/10.3390/molecules28166107/s1. Table S1: Principles, advantages and drawbacks of common radioactive surface decontamination methods; Figure S1: Chemical structures of (a) NS, (b) XG, (c) TD, (d) PA and (e) NDMP.

## Sample Availability

Samples of the compounds are not available from the authors.
