# Supplementary material for: Effects of Different Types of Stabilizers on the Properties of Foam Detergent Used for Radioactive Surface Contamination
Source: Molecules. 2023 Aug 17;28(16):6107. doi: 10.3390/molecules28166107 (PMC10458476; doi:10.3390/molecules28166107)
Supplement: Supplementary file 1 [file molecules-28-06107-s001.zip › molecules-2528193-supplementary.pdf]

# Supplementary Materials

# Effects of Different Types of Stabilizers on the Properties of Foam Detergent Used for Radioactive Surface Contamination

**Hao Zhang [1,2], Lili Liang [2], Hailing Xi [3,*], Xiaoyan Lin [2,*], Zhanguo Li [3] and Yu Jiao [1]**

[1] School of Science, Xichang University, Xichang 615013, China
[2] Engineering Research Center of Biomass Materials, Ministry of Education, School of Materials Science and Engineering, Southwest University of Science and Technology, Mianyang 621010, China
[3] State Key Laboratory of NBC Protection for Civilian, Beijing 102205, China;
* Correspondence: xihailing@sklnbcpc.cn (H.X.); linxiaoyan@swust.edu.cn (X.L.)

**Table S1**. Principles, advantages and drawbacks of common radioactive surface decontamination methods

| Decontamination method | Principles | Advantages | Drawbacks | Reference |
|---|---|---|---|---|
| Mechanical | Removal of surface contamination by wiping, scraping, suction, etc. | Easy to operate, simple equipment | Easy to cause surface damage, the solution produces radioactive aerosol, low decontamination rate | [6,7] |
| High-pressure water | Pressurized water is used to wash away soluble contaminants and some particles from the surface. | Unlimited surface shape, fast decontamination, and used for large area contamination | A good deal of radioactive waste liquid, easy to cause secondary pollution, and low decontamination rate | [6] |
| Laser | The laser beam vaporizes the contaminants by heat and separates them from the surface. | High decontamination rate, good accuracy and less radioactive waste | Generates radioactive aerosols, high decontamination costs, and usually used to decontaminate metal surface | [4,8] |
| Ultrasonic | Contaminants are removed from the surface by the cavitation and direct flow of ultrasound in the liquid. | Less restricted by surface shape, less damage to the surface and less chemical reagent consumption | Unsuitable for large area decontamination, and may cause damage to the bonding site | [9,10] |
| Reagent Washing | The solution containing chemical reagents such as acids, bases, oxidants and chelators is used to dissolve surface contaminants. | Less restricted by surface shape and high decontamination rate | Easy to corrode the surface, consume lots of chemical reagents and produce much waste liquid | [11,12] |
| Electrochemistry | Through the principle of electrolysis, the surface contaminants placed on the anode are dissolved in the electrolyte. | Less chemical reagent consumption and high decontamination rate | Not suitable for decontaminating large objects, usually used for metal surfaces, and expensive costs | [13] |
| Gel | The gel adheres to surface for a long time, and the pollutants enteres the gel for decontamination. | Decontamination is not limited by surface shape and can be used on inclined and vertical surfaces. | Complicated recovery of gels and low decontamination rate | [5,14] |
| Peelable Film | After the film-forming solution is sprayed onto the surface and dried, the contamination is adhered or absorbed into the peelable film. | No secondary pollution,and less radioactive waste | Long drying time to form film, complex peeling and recycling of the film, and expensive costs | [15,16] |

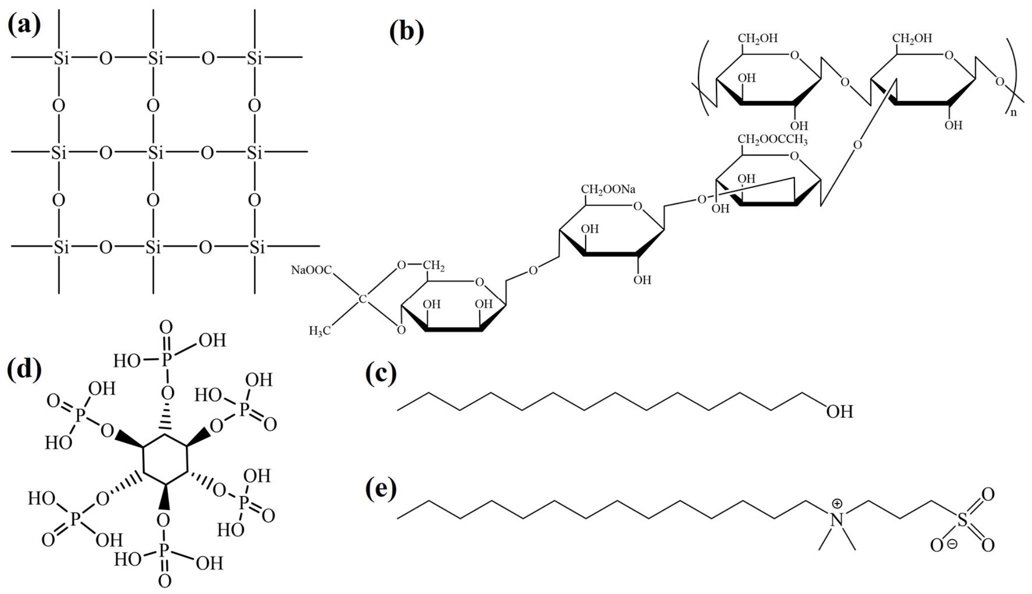


**Figure S1.** Chemical structures of (a)NS[20, 28], (b) XG[18], (c) TD[24], (d) PA[25] and (e)NDMP[24]
